# Adaptation and Exaptation: From Small Molecules to Feathers

**DOI:** 10.1007/s00239-022-10049-1

**Published:** 2022-03-04

**Authors:** Moran Frenkel-Pinter, Anton S. Petrov, Kavita Matange, Michael Travisano, Jennifer B. Glass, Loren Dean Williams

**Affiliations:** 1 NASA Center for the Origins of Life, Atlanta, GA 30332-0400 USA; 2 NSF-NASA Center of Chemical Evolution, Atlanta, GA 30332-0400 USA; 3 Institute of Chemistry, The Hebrew University of Jerusalem, 91904 Jerusalem, Israel; 4 School of Chemistry and Biochemistry, Georgia Institute of Technology, Atlanta, GA 30332-0400 USA; 5 Department of Ecology, Evolution and Behavior, University of Minnesota, Saint Paul, MN 55108 USA; 6 School of Earth and Atmospheric Sciences, Georgia Institute of Technology, Atlanta, GA 30332-0400 USA

**Keywords:** Exaptation, Evolution, Recursion, Chemical origins of life, Metabolites

## Abstract

Evolution works by adaptation and exaptation. At an organismal level, exaptation and adaptation are seen in the formation of organelles and the advent of multicellularity. At the sub-organismal level, molecular systems such as proteins and RNAs readily undergo adaptation and exaptation. Here we suggest that the concepts of adaptation and exaptation are universal, synergistic, and recursive and apply to small molecules such as metabolites, cofactors, and the building blocks of extant polymers. For example, adenosine has been extensively adapted and exapted throughout biological evolution. Chemical variants of adenosine that are products of adaptation include 2′ deoxyadenosine in DNA and a wide array of modified forms in mRNAs, tRNAs, rRNAs, and viral RNAs. Adenosine and its variants have been extensively exapted for various functions, including informational polymers (RNA, DNA), energy storage (ATP), metabolism (e.g., coenzyme A), and signaling (cyclic AMP). According to Gould, Vrba, and Darwin, exaptation imposes a general constraint on interpretation of history and origins; because of exaptation, extant function should not be used to explain evolutionary history. While this notion is accepted in evolutionary biology, it can also guide the study of the chemical origins of life. We propose that (i) evolutionary theory is broadly applicable from the dawn of life to the present time from molecules to organisms, (ii) exaptation and adaptation were important and simultaneous processes, and (iii) robust origin of life models can be constructed without conflating extant utility with historical basis of origins.

**Supplementary Information:**

The online version contains supplementary material available at 10.1007/s00239-022-10049-1.

## Introduction

Evolution is a dogged tinkerer (Jacob 1977), sculpting by adaptation and purloining by exaptation. Formally, adaptation tunes a trait or system over time, while exaptation co-opts an existing trait or system for new function. While the term exaptation was coined by Gould and Vrba in the early 1980s (Gould and Vrba 1982; Gould 2002), the concept was familiar to Darwin, who recognized that the swim bladder in fish was originally used for flotation, and was thereafter co-opted for a very different purpose: respiration (Darwin 1859).

Exaptation operates in diverse spaces. The fragile malleus and incus bones, used to transmit vibrations within the mammalian ear, were exapted from dense jaw bones of reptiles (Anthwal et al. 2013). The ears of elephants were co-opted for thermal regulation (Phillips and Heath 1992). Feathers, used for flight in birds, descended in the other direction, from thermally regulating structures (Prum 1999; Dhouailly et al. 2019; Pan et al. 2019).

Exaptation is ubiquitous in a broad variety of realms, including language (Williams 1983; Traugott 2004), music (Ryu 2010; Barthet et al. 2014; Youngblood 2019), and urban planning and architecture (Furnari 2011). Exaptation is critical to technological innovation (Andriani and Carignani 2014; Ferreira et al. 2020); co-option of proteins such as restriction enzymes, CRISPR-Cas, Taq polymerase, T7-RNA polymerase, and antibodies forms the basis of biotechnology. Microwave ovens use technology co-opted from World War II magnetrons (for RADAR) (Andriani and Cohen 2013).

**Fig. 1:**
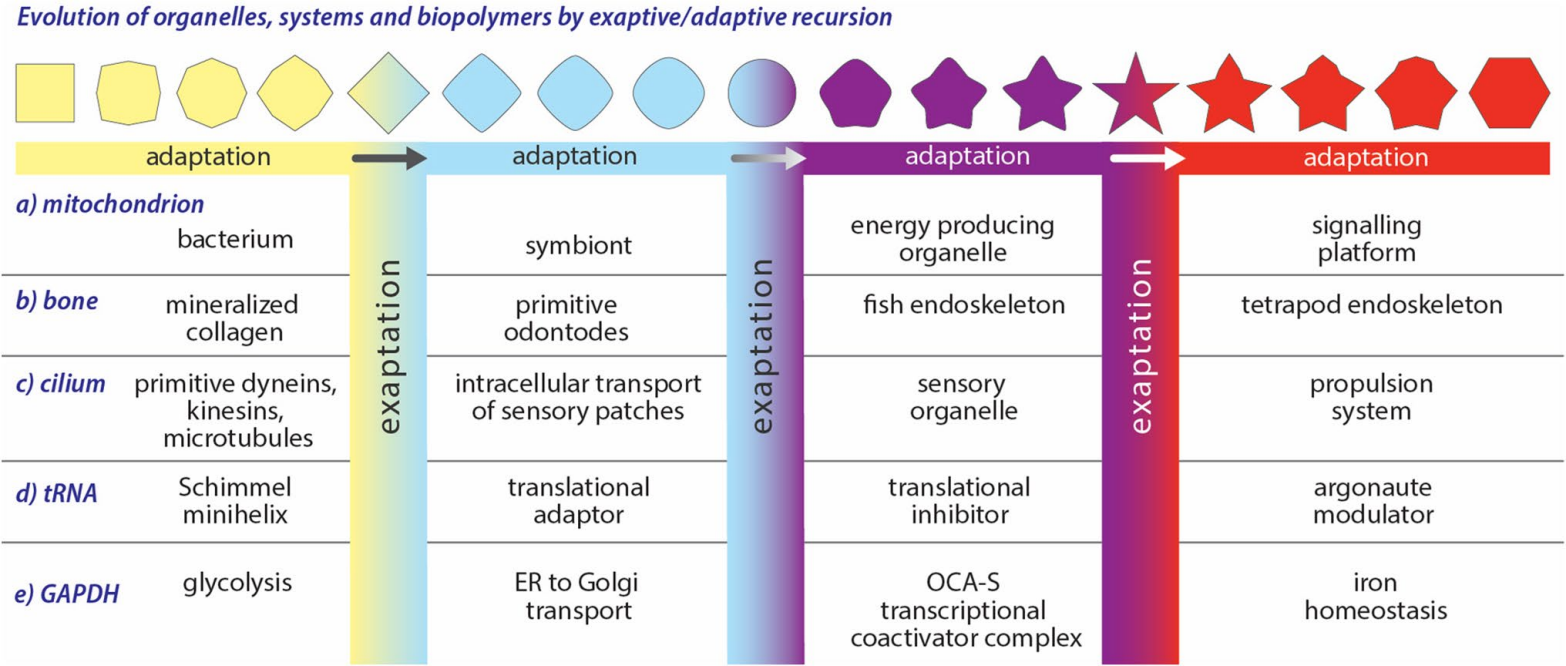
Schematic representation of exaptive/adaptive processes in which changes in shape represent adaptation, while changes in color represent exaptation. Exaptive/adaptive processes during evolution of (a) the mitochondrion, (b) bone, (c) the cilium, (d) tRNA, and (e) GAPDH. In several cases the specific ordering of the exapted/adapted species is tentative. This schematic is not intended to indicate that these processes share a common timeline or occur in a consequential order rather than in parallel. Both serial (recursive) and parallel exaptive/adaptive processes are included. The Schimmel minihelix is described in (Schimmel and de Pouplana 1995)

Here we suggest that in biology, exaptive and adaptive processes (i) are coupled and synergistic; these processes can radically accelerate each other, (ii) are prevalent on broad biological scales, from small molecules to organisms, (iii) have operated over deep time, from chemical evolution during the origins of life, to contemporary biology, and (iv) are recursive, endlessly creating, and relaunching from new landscapes. Our use of the phrase ‘exaptive/adaptive recursion’ (illustrated in Fig. 1) is consistent with Gould and Vrba who noted that feathers, initially adapted for thermoregulation, were serially exapted/adapted for flight and then to assist in catching prey (Gould and Vrba 1982; Gould 2002). Exaptive/adaptive processes can fork, meaning a trait and its ancestor can advance in parallel, exploring multiple new landscapes while preserving ancestral functions.

### Exaptation/Adaptation at the Organismal Level

Mitochondrial origins and evolution (Gray 2015; Roger et al. 2017; López-García et al. 2017) illustrate whole organism co-option, exaptive/adaptive recursion, synergism, and acceleration of rate of change. During this singular and profoundly consequential transformation, the ancestor of a α-proteobacterium was recursively exapted/adapted to ultimately form a eukaryotic organelle that is tightly integrated with the host (Fig. 1). In a serial process, the bacterium (i) physically entered the host cytosol, (ii) developed protein import systems for receiving retargeted proteins encoded in the host genome, (iii) developed small molecule transporters and carriers for retargeted metabolites, (iv) surrendered portions of its genome to the nuclear genome of the host, (v) remodeled its translation system but retained genes for mito-rRNAs and mito-tRNAs, and (vi) specialized and elaborated the organelle envelope and anchored to the cytoskeleton. It did not end there. The extant mitochondrion is a regulatory hub and has been co-opted to perform many functions in eukaryotic cells. In a variety of species, the mitochondrion has been further exapted/adapted to serve as optical lenses (Almsherqi et al. 2012; Gavelis et al. 2017). The mitochondrion demonstrates the synergism of exaptive/adaptive recursion; the extent and rate of change of the organism that were co-opted to form the mitochondrion far exceed those of α-proteobacteria in general (Roger et al. 2017; Petrov et al. 2019).

### Exaptation/Adaptation at the Sub-Organismal Level

Exaptive/adaptive recursion is a general phenomenon that operates at the level of systems (Fig. 1), illustrated here by the evolution of mammalian bone. An early step in vertebrate mineralization was an exaptation of collagen to mineralized odontodes for catching and crushing prey (Doherty et al. 2015). These mineralized structures were exapted/adapted as an aquatic exoskeleton and/or aquatic endoskeleton, which was exapted/adapted as a terrestrial endoskeleton. The fins of fishes were repurposed as tetrapod limbs; bones in a human arm, wrist, and hand or the in the wing of a bat can be mapped to ancestral bones in the fin of a fish (Clack 2009; Nakamura et al. 2016). In additional exaptation/adaptations, bone has been recruited to store and regulate calcium and phosphate. In humans, elevated estrogen promotes skeletal calcium sequestration in bone that is reversed during pregnancy and lactation (Järvinen et al. 2003). Forking, the parallel branching of adaptive/exaptive processes into multiple landscapes is illustrated by the proposal of the common evolutionary origins of odontodes, teeth, dermal scales, and bones (Dhouailly et al. 2019).

The evolution of cilia, used to propel unicellular eukaryotic microbes, is another example of exaptive/adaptive recursion of biological systems. Cilia are thought to have ancestry in transport systems for membrane sensory patches. These sensory patches were exapted for use as extruded sensory organelles that were re-exapted for propulsion (Beeby et al. 2020).

### Exaptation/Adaptation of Macromolecules: Proteins and RNAs

Exaptation is possibly even more rampant at levels of individual biopolymers than at higher biological levels. Biopolymers can be exapted by a variety of mechanisms, including neofunctionalization (Rastogi and Liberles 2005) and moonlighting (Mani et al. 2015; Singh and Bhalla 2020). In neofunctionalization, one paralog of a duplicated gene takes on new functions that are facilitated by change of sequence. A moonlighting protein switches function without change of sequence. In these cases, adaptation consists of changes in location, level of expression, or association with ligands or other biopolymers. tRNA, the universal translational adapter, has been recursively exapted and adapted, while maintaining the ancestral adapter function (Fig. 1, Table 1). In eukaryotic systems, tRNA has been co-opted for a broad variety of functions.

**Table 1:** Exaptation of proteins and RNAs

	Ancestral function^a^	Exapted function(s)
*Protein*		
Cytosolic ribosomal proteins (Wang et al. 2015; Lu et al. 2015)	Structure and assembly of the ribosome	In transcription, cell growth and proliferation, apoptosis, mRNA splicing, DNA repair, cellular development, and cellular differentiation
Ribonuclease III (Petrov et al. 2019)	Cleavage of double-stranded RNA	As a mitochondrial ribosomal protein
MutT (Petrov et al. 2019)	Repair of DNA containing 8-oxoguanine	As a mitochondrial ribosomal protein
Aminoacyl-tRNA synthetases (Guo and Schimmel 2013)	Covalently link amino acids to their cognate tRNAs	In metabolism, development, angiogenesis, tumorigenesis, immune response, neuronal function, and inflammation
Glyceraldehyde-3-phosphate dehydrogenase (GAPDH) (Singh and Bhalla 2020)	Catalyzes the sixth step of glycolysis, the conversion of d-glyceraldehyde 3-phosphate to 3-phospho-d-glyceroyl phosphate	In apoptosis, iron transport, membrane fusion, transcriptional regulation, vesicle transport, and cellular response to oxidative stress and hypoxia
Argininosuccinate lyase (Piatigorsky 2003; Gavelis et al. 2017)	Catalyzes the fourth step of the urea cycle and is involved in the biosynthesis of arginine	Light focusing δ-crystallins in birds and reptiles
Lactate dehydrogenase (Hendriks et al. 1988)	Catalyzes the reversible conversion of pyruvate to lactate and of NADH to NAD^+^	Light focusing ε-crystallins in birds and reptiles
Pancreatic trypsinogen (Chen et al. 1997)	A zymogen of trypsin, a digestive protease	An antifreeze protein in cold water fishes
Transposase (Fugmann 2010)	Cut-and-paste transposition	A V(D)J recombinase, which rearranges immunity-related genes
Protein of an endogenous retrovirus (Cornelis et al. 2015)	Retrovirus envelope formation	A mediator of placentation in mammals
*RNA*		
tRNAs (Luchetti and Mantovani 2013; Robeck et al. 2016)	Translational adaptors	BC1 and many additional short interspersed nuclear elements (SINEs) in animals with many functions
tRNA^val^ and tRNA^Phe^ (Greber et al. 2014; Rorbach et al. 2016; Brown et al. 2017)	Translational adaptors	Replacements for 5S rRNA in mammalian mitochondrial ribosomes
tRNA fragments (Schorn and Martienssen 2018; Magee and Rigoutsos 2020)	Translational adaptors	Guides to silencing of mRNAs by binding to argonaute and PIWI proteins, inhibitors of retrotransposition by binding to the primer binding site of LTR-retrotransposons, inhibitors of replication of retroviruses and LTR-retroelements, inhibitors of translation, and mediators of epigenetics
Dormant transposon (Ellis et al. 2018; Brosius 2019)	Transposon	Post-transcriptional regulation

^a^In several cases the characterization of ancestral versus exapted/adapted functions (i.e., the polarity) is tentative. Ancestry is relative to more recent exapted functions and should not be interpreted to imply ultimate ancestry

### Exaptation/Adaptation of Small Molecules

**Fig. 2:**
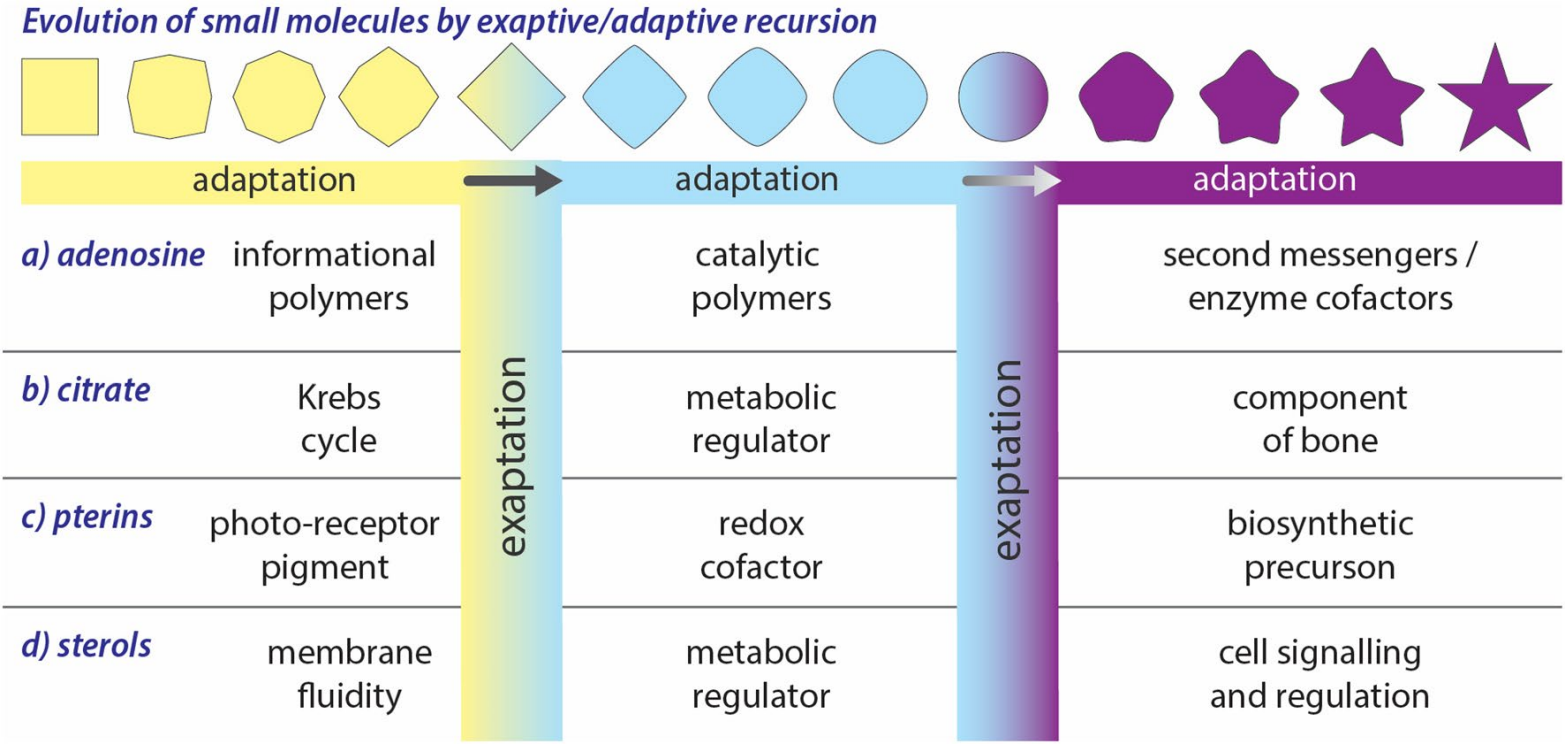
Schematic representation of exaptation/adaptation in which changes in shape represent adaptation, while changes in color represent exaptation. Exaptation/adaptation of (a) adenosine, (b) citrate, (c) pterins, and (d) sterols. The specific ordering of some exapted/adapted species (i.e., the polarity) is tentative (Color figure online)

**Fig. 3:**
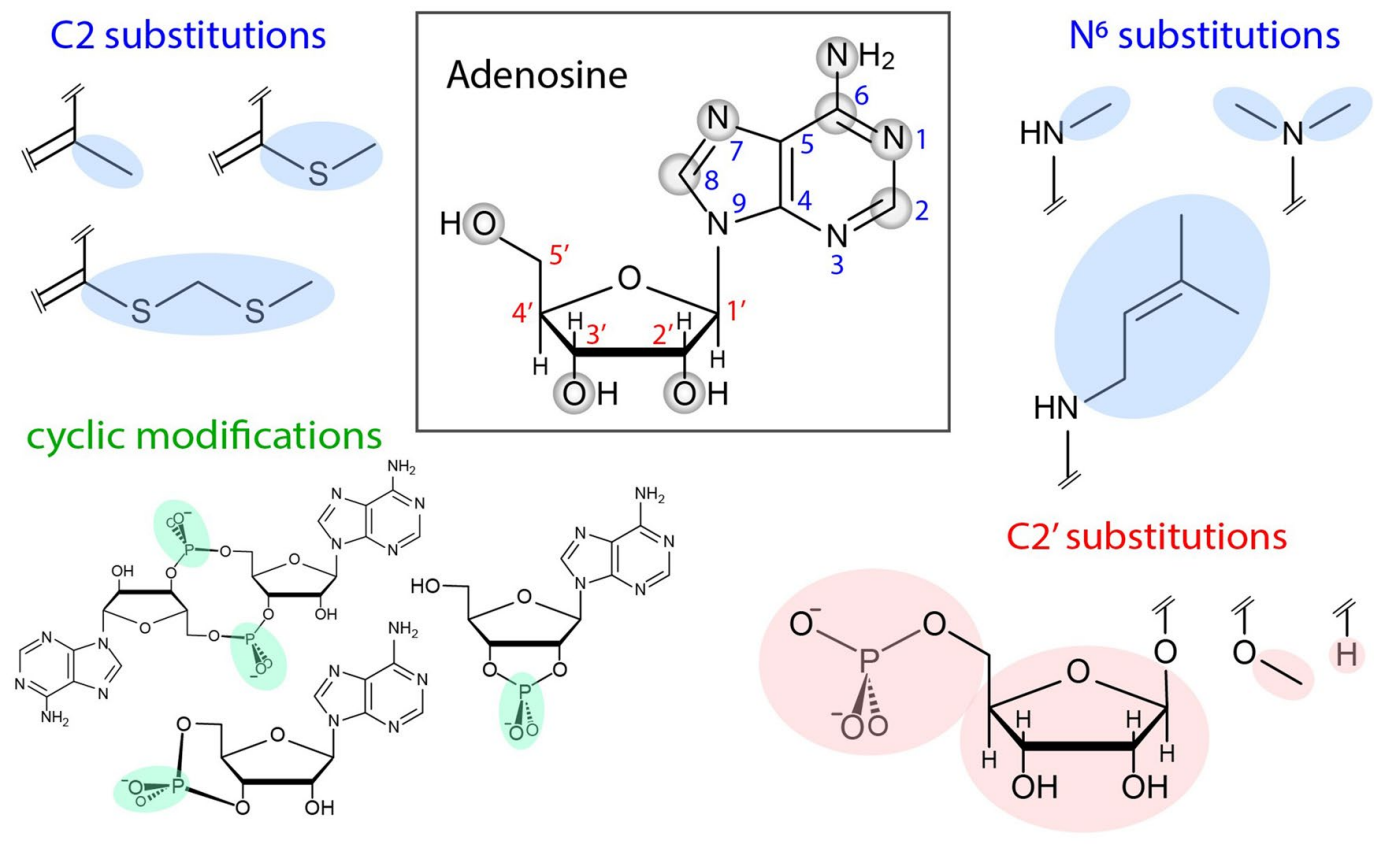
Adenosine (top center) is highly sculpted by adaptation and exaptation. Sites of chemical modification of adenosine are highlighted. Shown here are examples of the large number of chemical variants of adenosine found in various biological systems. Adenosine variants include inosine (Paul and Bass 1998), 1-methyl adenosine, 2-methyl adenosine, 6-methyl adenosine, 6-dimethyl adenosine, 7-methyl adenosine, 8-methyl adenosine (Demirci et al. 2010; Saikia et al. 2010; Motorin and Helm 2011; Liu and Pan 2016; Kanazawa et al. 2017), 6-isopentenyl adenosine (hydroxylated and unhydroxylated), a variety of 2-thiomethylated adenosine variants (Hoburg et al. 1979; Motorin and Helm 2011), 6-glycinylcarbamoyl adenosine, cyclic 6-threonylcarbamoyl adenosine and 2′-O-methyl adenosine (Gonzales-van Horn and Sarnow 2017), 2′-deoxyadenosine, and 2′-O-ribosyladenosine (phosphate) (Desgrès et al. 1989). Cyclic variants include 2′, 3′ cyclic adenosine phosphate, 5′, 3 cyclic adenosine phosphate, and 5′, 5′ cyclic di-adenosine phosphate

Do adaptation and exaptation operate at even more microscopic levels, at the levels of small molecules, such as metabolites, cofactors, or polymer building blocks? Small molecules are products of gene products and are less proximal to genotype. Yet, these building blocks, like organisms, organs, proteins, and RNAs, undergo changes in structure and function over the course of evolution. As illustrated schematically in Figs. 2 and 3, small molecules can be chemically sculpted to tune function, in analogy with changes in sequences of proteins and RNAs. Moreover, molecules that serve one function are frequently co-opted in the absence of sculpting to serve other functions, in analogy with protein moonlighting. Protein and small molecule exaptation are coupled. When a protein has been exapted into a new functional space, complementary changes are imposed at the level of small molecule effectors and substrates.

Adenosine is a remarkable example of adaptive/exaptive recursion at the level of small molecules (Fig. 3). Adenosine is a component of RNA, ATP, coenzyme A, NAD and FAD, cAMP, and cyclic diAMP. In bacteria, cAMP is a positive regulator of the *lac* operon (De Crombrugghe et al. 1984). In eukaryotes, cAMP activates protein kinase A, leading to phosphorylation of ion channels and transcription factors (Hanks and Hunter 1995). In primates, adenosine moonlights as a neurotransmitter (Ciruela et al. 2006). In starfish, 1-methyladenine is a hormone (Kanatani and Hiramoto 1970).

Adenosine has been intensively adapted, by chemical sculpting, to form a large group of chemical variants (Boccaletto et al. 2018; Hernández-Morales et al. 2019). Chemical variants of adenosine include 2′ deoxyadenosine, nicotinamide adenine dinucleotide, flavin adenine dinucleotide, S-adenosylmethionine, acetyl CoA, and the 5′ adenosyl radical in radical SAM enzymes (Frey et al. 2008) and vitamin B12 (Kräutler 2012). Phosphorylated derivatives of adenosine include 5′ mono, di- and tri- phosphates, 2′ adenosine monophosphate, cyclic 2′, 3′ adenosine phosphate, cyclic 5′, 3 adenosine phosphate, and cyclic diAMP. Adenine base modifications are immense in number and variety. A representative survey of adenosine variants is shown in Fig. 3.

The exaptation/adaption of small molecules is not limited to adenosine and appears to be important in small molecule biochemistry in general (Table 2). Citrate, an intermediate in the Krebs cycle, has been widely exapted/adapted (Fig. 2) (Iacobazzi and Infantino 2014; Williams and O’Neill 2018; Granchi et al. 2019) and is critical in sterol and fatty acid biosynthesis, metabolic regulation, as a component of bone, and as mediator of inflammation. Guanosine, a building block of RNA, has also been widely exapted/adapted (Mellion et al. 1981; Frizzo et al. 2001); tunable pigment cells called guanophores reflect light from crystalline guanine (Fudouzi 2011). Molybdopterin has been exapted/adapted repeatedly and has been incorporated into a broad variety of enzymes (Leimkühler and Iobbi-Nivol 2016), including xanthine oxidase, DMSO reductase, sulfite oxidase, nitrate reductase, ethylbenzene dehydrogenase, glyceraldehyde-3-phosphate ferredoxin oxidoreductase, respiratory arsenate reductase, carbon monoxide dehydrogenase, and aldehyde oxidase. Gamma-aminobutyric acid (GABA), a mediator of stress response in bacteria, has been exapted as an inhibitory neurotransmitter in vertebrates (Moore and Speh 1993) and a signaling molecule and metabolite in plants (Bouche and Fromm 2004).

**Table 2:** Exaptation of small molecules

Molecule	Ancestral function^a^	Exapted function(s)
Adenosine (Kanatani and Hiramoto 1970; De Crombrugghe et al. 1984; Hanks and Hunter 1995; Ciruela et al. 2006)	Component of RNA	Energy source (ATP, coenzyme A), redox cofactor (NAD, FAD), regulator of the *lac* operon, activator of protein kinase A, neurotransmitter, and hormone
Citrate (Iacobazzi and Infantino 2014; Williams and O’Neill 2018; Granchi et al. 2019)	Intermediate in the Krebs cycle	Fatty acid biosynthesis, sterol biosynthesis, metabolic regulation, metal coordination, component of bone, inflammation, insulin secretion, histone acetylation, prostatic cell function, and carbon source (anaerobic bacteria)
Beta-carotene (Pryor et al. 2000; Dieser et al. 2010; Cazzonelli 2011; Kirti et al. 2014)	Survival in cold environments	Precursor of Vitamin A, light-harvesting pigment, photo-protection, glycoprotein synthesis, protection from oxidation, and pollinator attractant
Riboflavin (Rajamani et al. 2008; Dakora et al. 2015; Sepúlveda Cisternas et al. 2018)	Electron transport	Reduction of glutathione, production of pyridoxic acid, α-ketoglutarate, branched-chain amino acids, and fatty acids, oxidation of pyruvate, conversion of retinol to retinoic acid bacterial pigment, coenzyme (vitamin B2), anti-oxidant inducer (plants), induction of disease resistance (plants), and quorum sensing (AHL mimic)
Pterins (Basu and Burgmayer 2011)	Pigments	Electron transfer cofactors, redox cofactors, precursor of folates, and toxins
GABA (Gamma-aminobutyric acid) (Moore and Speh 1993; Bouche and Fromm 2004; Feehily and Karatzas 2013)	Stress response in bacteria	Inhibitory neurotransmitter in vertebrates, and signaling molecule and metabolite in plants
Sterols (Gil et al. 2018), (de Jong et al. 2003)	Membrane fluidity	Metabolite precursors, growth regulator (plants), calcium absorption (Vitamin D), transcription regulator (progesterone), and cell signaling
Guanosine (Mellion et al. 1981; Frizzo et al. 2001)	Component of RNA	Glutamate regulator, inhibitor of platelet aggregation, second messenger, alarmone in bacteria, and energy source for translation
Lactate (Sola‐Penna 2008; Proia et al. 2016)	Energy source (fermentation)	Signaling molecule (brain), muscle glycogen production, and spermatogenesis

^a^The term “function” here is equivalent to “character” in reference (Stevens 1980). Ancestry is relative to more recent exapted functions and should not be interpreted to imply ultimate ancestry

The results of our survey of small molecule structure and function (Table 2) make clear that small molecules are broadly sculpted and repurposed, undergoing exaptation and adaptation in analogy with RNAs, proteins, and macroscopic biological structures. The wide variety of functional roles of a given small molecule, extensive sculpting of given chemical frameworks, and the specific phylogenetic localization of some functions are consistent with adaptation and co-option.

### Exaptive/Adaptive Recursion and the Reconstruction of History

Gould and Vrba noted that exaptation obscures history. As stated by Gould, “hardly any principle in general historical reasoning (not only in evolutionary theory) can be more important than clear separation between the historical basis of a phenomenon and its current operation” (Gould 2002). We formalize that guideline to say that the intense creativity of evolutionary processes obscures and disguises both historical events and, to a lesser extent, ahistorical principles (Pascal et al. 2013). Exaptive/adaptive recursion opens new phenotypic landscapes from which it launches new rounds of exaptive/adaptive recursion. Features of ancestral landscapes are not necessarily shared by or communicated to progeny landscapes. Exaptive/adaptive recursion creates “function horizons” that can obstruct the inference of history. In examples of function horizons, binding of tRNA fragments to argonaut to silence mRNAs, a contemporary function, is a poor guide to the deep history of tRNA. The use of feathers for flight should not be taken to indicate flying was a selected trait during the origins of feathers. Mitochondrial lens are functionally unrecognizable as descendants of the bacterial ancestors. Inspection of a microwave oven does not reveal the protagonists or the victors of the Battle of Britain. Darwin too understood that endless repurposing means that extant structure and function can be a misleading guide to origins: “Thus throughout nature almost every part of each living being has probably served, in a slightly modified condition, for diverse purposes and has acted in the living machinery of many ancient and distinct specific forms” (Darwin 1859) (page 284).

### Exaptive/Adaptive Processes and the Origins of Life

Exaptive/adaptive recursion is explicitly incorporated into some models of the origins of life. Noller (Noller 2012) and Cech (Cech 2009) each proposed that amino acids and peptides were initially selected by their abilities to enhance RNA function. RNA-binding was a selected trait of proto-protein (non-coded, heterogeneous oligomeric ancestors of extant protein) conferring advantage by increasing accessible structural and functional space of RNA. Translating the Noller/Cech model to our vernacular, we would say that molecules initially selected for RNA-binding have been recursively exapted and adapted, ultimately yielding extant non-RNA-related functions as enzymes, fibers, channels, and compartments.

Many models of the origins of life, some of them broadly accepted, use extant function to explain history. These models, in our view, fail to appreciate function horizons and the vast creative capacity of evolution. In fact, many origin of life models specify a function or trait in contemporary life and treat it as a requisite for the origin of life (Lanier and Williams 2017). A function or trait is excised from extant biological context and relocated in time, space, and environment to the ancient Earth.

In RNA World models (Jeffares et al. 1998; Ricardo et al. 2004; Orgel 2004; Robertson and Joyce 2012; Higgs and Lehman 2015; Vázquez-Salazar and Lazcano 2018), ancestral functions of RNA, including catalysis of chemical reactions, storage of information, and utility of a monomeric metabolites, have been maintained, from the chemical origins of life to extant biology (Table S1). In these models, RNA appears to be privileged (Lanier and Williams 2017) and is exempted from exaptive/adaptive recursion. Benner, for example, writes that contemporary ribosomes, ribozymes, and metabolism are evidence for an ancient RNA World (Ricardo et al. 2004; Neveu et al. 2013). The discovery of extant catalytic RNAs by Cech (Kruger et al. 1982) and Altman (Guerrier-Takada et al. 1983) directly inspired Gilbert’s influential ‘Origin of Life: The RNA World’ (Gilbert 1986).

We suggest that exaptive/adaptive recursion was as important during prebiotic chemical evolution and early biology as it is in extant biology, and that RNA was not exempt. If so, functions of RNAs and ribonucleotides in extant biology, including catalysis, information, and metabolism may not have been functions of a historical proto-biopolymers (Pascal et al. 2013). We suggest that mutual protection from hydrolysis, promoted by assembly and co-assembly (Runnels et al. 2018; Frenkel-Pinter et al. 2020), may have driven co-evolution of proto-polynucleotide and proto-polypeptide during wet-dry chemical evolution. Assembly was exapted for catalysis. In this model, the extant functionalities of both RNA and protein are derived, not ancestral; a subset of oligomers or polymers with sophisticated assembly and co-assembly properties was exapted/adapted for extant catalytic, informational, and metabolic functions. Our analysis of the ribosome (Kovacs et al. 2017; Bowman et al. 2020) supports this model; intrinsically disordered proto-polymers that associated with RNA were co-opted to form β-hairpins, which were co-opted to form β-domain folds, which were co-opted to form complex folds containing α-helices.

We believe that the machine that converted a bacterium to a mitochondrion to an optical lens unescapably exapted and adapted building blocks and proto-polymers. Hence, the long history of exaptive/adaptive recursion during chemical and biological evolution should challenge the direct utility of extant catalytic, informational, and metabolic functions of RNA as critical traits during the origins of life.

### Constructive Neutral Evolution (CNE)

The framework here is consistent with adaptive rationales, but does not exclude models such as CNE (Stoltzfus 1999; Muñoz-Gómez et al. 2021). CNE describes a multi-step process in which neutral, non-adaptive change opens capacities for complementation and co-dependency. In a first CNE step, an intrinsic or environmental change would alter part of a redundant pathway to produce a modified molecule with little or no immediate function. In subsequent steps, other changes would stumble upon functions for this modified molecule, ratcheting the complexity. A CNE model is consistent with the observation of extensive molecular diversification of a given molecular framework. The sculpting of adenosine, for example, that produced an enormous number of interdependent chemical variants, could have taken place in the absence of positive selection. It seems likely that CNE operates at levels of small molecules, as a molecular search engine for new chemistries and new entities.

Here, we use terms “function” (of biopolymers) or “chemical variant” (of small molecules) to describe a trait or property that is heritable and subject to exaptation/adaption. These terms are roughly equivalent to the more traditional “character” used in cladistics (Stevens 1980; Nixon and Carpenter 2012). Polarities (i.e., chronologies) of exaptation/adaptation of various functions and chemical variants have been inferred in a variety of ways. We use qualitative parsimony to establish polarities of macromolecules and small molecules. In general, ancestral characters are universal to archaea and/or bacteria and derived characters are specific to eukarya. The polarities of changes of mitochondrion, bone, the cilium, tRNA, and GAPDH in Fig. 1 are established in the literature. Polarity reversals or ambiguities do not alter the fundamental conclusions about exaptative/adaptive recursion. In several cases the polarities are tentative.

## Supplementary Information

Below is the link to the electronic supplementary material.

Supplementary file1 (DOCX 19 kb)
