# Supplementary material for: Adaptation and Exaptation: From Small Molecules to Feathers
Source: J Mol Evol. 2022 Mar 4;90(2):166–75. doi: 10.1007/s00239-022-10049-1 (PMC8975760; doi:10.1007/s00239-022-10049-1)
Supplement: Supplementary file 1 — Supplementary file1 (DOCX 19 kb) [file 239_2022_10049_MOESM1_ESM.docx]

# Table S1. Use of Extant Function to Explain History in the Context of the RNA World.^a,b^

| “…the primary evidence for an RNA world comes from the roles of RNA in modern cells.” - Jeffares (1998) (Jeffares et al. 1998)  “The general notion of an “RNA world” is that, in the early development of life on the Earth, genetic continuity was assured by the replication of RNA, and RNA molecules were the chief agents of catalytic function.” - Joyce and Szostak (2018) (Joyce and Szostak 2018)  “The first stage of evolution proceeds, then, by RNA molecules performing the catalytic activities necessary to assemble themselves from a nucleotide soup. The RNA molecules evolve in self-replicating patterns, using recombination and mutation to explore new functions and to adapt to new niches.” - Gilbert (1986) (Gilbert 1986)  “The… properties of RNA molecules… combined with their catalytic activities and ubiquity in cellular processes, suggest that during an early, perhaps primordial, stage RNA molecules played a much more conspicuous role in heredity and metabolism.” - Lazcano (2018) (Vázquez-Salazar and Lazcano 2018)  “The central role of RNA in protein translation and RNA splicing, together with a diverse array of different functional RNAs such as ribozymes, riboswitches, tRNA, mRNA, ncRNAs and other regulatory RNAs found to different extents in all domains of life, provide compelling support for a central role of RNA in early biology.” - Holliger (2017) (Wachowius et al. 2017)  “RNA‐based systems are attractive because inheritance and function can be embodied within the same class of molecules.” – Szostak (2017) (Szostak 2017) |
| --- |
